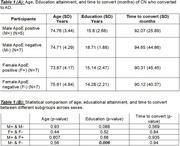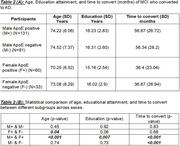# Sex but not ApoE status predicts conversion to dementia in married individuals

**DOI:** 10.1002/alz70860_103444

**Published:** 2025-12-23

**Authors:** Rishitha Praveen, Anshita Rathore, Virendra R Mishra

**Affiliations:** ^1^ Jawaharlal Nehru National College of Engineering, Shivamogga, Karnataka, India; ^2^ Gandhi Medical College, Bhopal, Madhya Pradesh, India; ^3^ University of Alabama at Birmingham, Birmingham, AL, USA

## Abstract

**Background:**

The interplay of genetic, social, and educational factors in influencing the risk of dementia remains a topic of considerable interest. Female sex, education, and the apolipoprotein‐E (ApoE) gene, particularly its ε4 allele, are well‐established risk factors for dementia, but its interaction with non‐genetic determinants like marital status is less understood. This study investigates and compares the likelihood of conversion to dementia in married male and female ApoE (+) and ApoE (‐) individuals to identify the most predictive risk factor among sex, education, and ApoE status for converting to dementia in married individuals.

**Method:**

Data from the Alzheimer's Disease Neuroimaging Initiative (ADNI) were utilized to assess progression to dementia in individuals transitioning from cognitively normal (CN) or mild cognitive impairment (MCI) to dementia due to Alzheimer's disease (AD). Individuals were categorized based on ApoE genotype, sex, and marital status. Statistical analyses examined conversion rates across these subgroups, with a particular focus on how age and education influence the progression to dementia in various subgroups in married individuals.

**Result:**

CN to AD (Table 1): In the CN to AD cohort, education significantly differed between ApoE (‐) sexes, with males exhibiting higher education levels (*p* = 0.0061). However, no significant differences were found in time to conversion (*p* = 0.94) or age (*p* = 0.56) for these groups. In comparisons between male and female ApoE (+) individuals, no significant differences were noted in age (*p* = 0.807), education (*p* = 0.68), or time to conversion (*p* = 0.935). Similarly, no significant differences emerged between ApoE (+) and ApoE‐negative groups within males and females regarding age, education, or time to conversion.

MCI to AD (Table 2): Female ApoE (+) individuals demonstrated significantly faster conversion to AD compared to their male counterparts. Notable significant (*p* <0.05) differences were observed in age, education, and time to conversion. Among females, age significantly influenced dementia risk for ApoE (+) individuals (*p* = 0.03855), while education acted as a protective factor for ApoE (‐) females (*p* = 0.0061).

**Conclusion:**

Our preliminary findings suggest that sex is the strongest predictor of converting to AD from MCI, followed by age and education in married individuals regardless of their ApoE status. Future studies will compare volumetric MRI to better understand their neuroanatomical differences.